# Multipath: An R Package to Generate Integrated Reproducible Pathway Models

**DOI:** 10.3390/biology9120483

**Published:** 2020-12-21

**Authors:** Zaynab Hammoud, Frank Kramer

**Affiliations:** IT-Infrastructure for Translational Medical Research, University of Augsburg, 86159 Augsburg, Germany; frank.kramer@informatik.uni-augsburg.de

**Keywords:** multilayer networks, data integration, biological pathways, reproducibility, visualization

## Abstract

**Simple Summary:**

In biological terms, the term "pathway" is used to describe a collection of processes within a cell that lead to one or more actions. The graphical representation of these processes enables the reader to understand complex relationships and interactions much more easily compared to free-text descriptions. While there is usually agreement on the existence and function of these high-level processes, the specific molecules and their interactions are often disputed and a matter of current research. A standardized computational representation of biological networks has become desirable, especially with the recent surge in new knowledge generation in biology and medicine. Our work is influenced by challenges emerging from previous work on biological pathways, knowledge encoding, and visualization as well as pathway databases. Our main motivation is the difficulty of reproducing pathway knowledge used within publications, even in top-tier journals. We propose a new way of integrating and modeling pathways and other influencing knowledge, such as drugs, and documenting their modifications using multilayered networks. We provide a tool that transforms encoded pathway data to multilayered graphs, with the possibility to modify them, and integrate other knowledge from external databases.

**Abstract:**

Biological pathway data integration has become a topic of interest in the past years. This interest originates essentially from the continuously increasing size of existing prior knowledge as well as from the many challenges scientists face when studying biological pathways. Multipath is a framework that aims at helping re-trace the use of specific pathway knowledge in specific publications, and easing the data integration of multiple pathway types and further influencing knowledge sources. Multipath thus helps scientists to increase the reproducibility of their code and analysis by allowing the integration of numerous data sources and documentation of their integration steps while doing so. In this paper, we present the package Multipath, and we describe how it can be used for data integration and tracking pathway modifications. We present a multilayer model built from the Wnt Pathway as a demonstration.

## 1. Introduction

Deciphering the inner workings of cells fascinates researchers all over the world. However, these processes are highly complex and heterogeneous, with countless players constantly interacting via biochemical reactions, signaling cascades and feedback loops. Knowledge about these processes can be organized into so-called pathways by grouping sets of interactions, which share a common goal or function [1]. Over the course of the last decades, an enormous amount of knowledge on molecular interactions within cells has been accumulated, with a plethora of methods and algorithms using this knowledge. Consequently, two problems are currently faced: the integration of the different pathways’ types and the irreproducibility of pathway illustrations used within journals.

Our aim is to provide a generic modelling framework to integrate multiple pathway types and further knowledge sources influencing these pathways. This framework is defined by a multi-layered model allowing automatic pathway transformations and documentation. By providing a tool that generates this model, we aim to facilitate the data integration, boost the reproducibility and increase the interoperability between different sources and databases in the field of pathways. The transformation of the pathway models helps structuring and condensing knowledge in specific areas of interests, e.g., removing layers with irrelevant information, coupling expression and pathway knowledge, and connecting drug-target and mutation layer connections, while documenting every step of this process. 

In this paper, we present the R package Multipath that creates these multilayer extendable models from BioPAX-encoded pathway files [2], and extract influencing knowledge from external databases. We show the multilayer model and the views that we generated from the Signaling by Wnt Signaling pathway.

This package uses freely available data sources like Reactome or other ontology-encoded pathway databases, using our R Packages rBiopaxParser [3] and mully [4]. Furthermore, we integrate open source tools like dbparser [5] and UniProt.ws [6] to retrieve information from the databases DrugBank [7] and UniProt [8].

## 2. Materials and Methods

Multipath is an R package that aims at creating reproducible pathway models. It allows the user to transform BioPAX encoded pathways into multilayered graphs, using the R packages mully [4] and rBiopaxparser [3]. Mully is an R package that allows the creation, modification, and visualization of multilayered graphs [4], while rBiopaxparser is an R package to parse, modify, and visualize pathway data encoded in the BioPAX standard [3]. It allows the user to parse the data and create a monolayered graph from it. The package mully is then used to transform this monolayered graph into a multilayered mully model. The elements of the pathways—which represent the nodes—are divided into groups based on their class, for example complexes, proteins, DNAs, RNAs, small molecules, etc. Each group of nodes is embedded into one layer of the resulting graph. The edges connecting the nodes are the interactions extracted from the BioPAX file.

**Figure 1:**
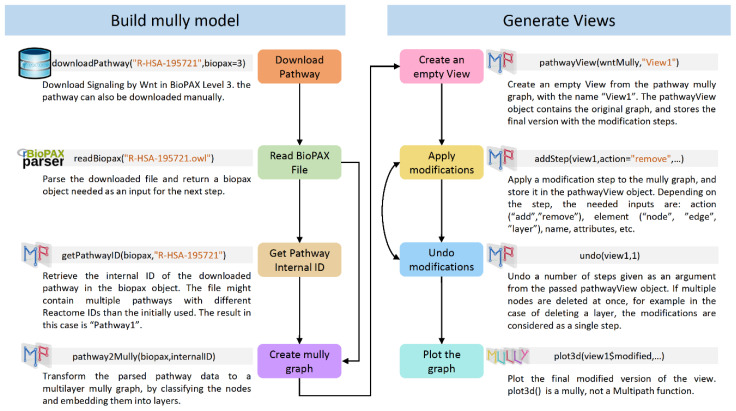
Reproducibility Workflow Diagram. The diagram shows the steps that have to be followed to generate reproducible multilayer pathway models. The Signaling by Wnt pathway was used as an example.

To build the BioPAX mully model, the user has to follow the following steps (Figure 1):

1. Read the BioPAX file using the rBiopaxparser function readBiopax() and provide the path to the BioPAX file containing the pathway information as an argument. The file can be downloaded manually or using the function downloadPathway().
2. Fetch the pathway’s internal ID using the function getPathwayID() from the BioPAX object returned in step 1 which might contain multiple pathways.
3. Provide the pathway’s ID alongside the BioPAX object as arguments to the Multipath function pathway2Mully() which returns the mully graph created from the parsed pathway information in the BioPAX object.

The processing time depends on the size of the BioPAX file being imported. A larger pathway may require a larger memory space and a longer time to be parsed, and transformed into a mully object.

We currently offer the function downloadPathway(), to download a pathway from the database Reactome [9] in BioPAX level 2 and 3. However, the user can download the pathways manually. A list of available repositories for pathways encoded in the BioPAX standard can be found in the pathway resource list Pathguide [10].

The generated mully graph is modifiable and retraceable. All modifications applied to the model can be stored in a view object and tracked using the feature track and undo. The view object contains different information concerning the modifications, including the timestamp of creation and last modification, the original graph and its final modified version, as well as the list of applied steps. 

To track the modification, the user has to call the function addStep(), to which a list of arguments should be provided: the action (add or remove), the element (node, edge or layer), the name of the element, and more arguments depending on the type of the element. It returns the view with the added steps. Removing a layer results removing a list of nodes and edges, which are all considered a single step.

The function undo(), which reverses the latest changes applied to the graph, requires the view and the number of steps to undo. The steps are reversed based on their id in the view. For instance, undoing a deletion of a node, will re-add the node and all its connections, and of a layer will re-add the layer, the nodes of this layer, and all their connections.

Multipath also offers data integration functions to extract any additional information needed from DrugBank [7] and UniProt [6]. These functions, that need the list of proteins’ and drugs’ IDs respectively as input, return integrated information existing in both databases. 

Functions querying other databases such as ChEBI [11] and PubChem [12] using the R Package webchem [13], KEGG [14], and ENSEMBL [15] are also being implemented and will be available soon.

## 3. Results

### 3.1. Pathway Data Integration

We present functions in Multipath that fetch information from external databases. Essential information on each node are taken from the original database of the given IDs; for example, information on proteins with UniProtKB/Swiss-Prot IDs are extracted from UniProt. The interactions are proved from different databases, and the source of the interactions added to the graph is assigned as an attribute to the edges. 

#### 3.1.1. DrugBank

**Figure 2:**
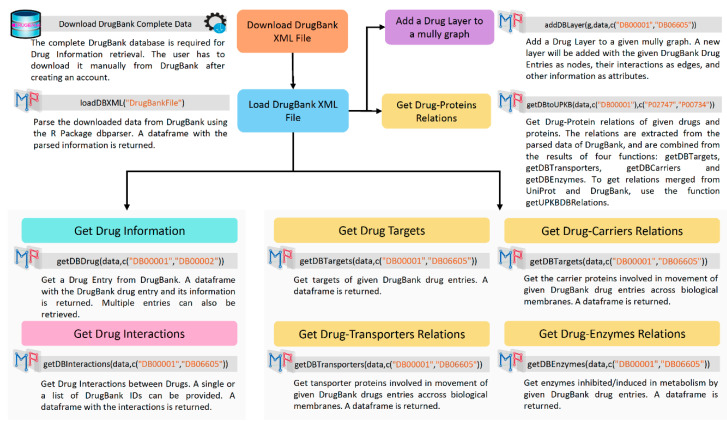
DrugBank Workflow Diagram. The Diagram shows the steps to get Drug Information from DrugBank using Multipath and the R Package dbparser.

DrugBank is an online freely accessible database for drug data and drug products [7]. The complete data can be downloaded from the official DrugBank website [16] in the XML format. To parse this data, the function loadDBXML() uses the R package dbparser [5]. This function should be called before using any other function related to DrugBank, since it returns the object containing the parsed information, and needed as an argument in all the related functions (Figure 2). The parsed data contain information on the drugs, as well as interactions between the drugs and between drugs and proteins. The latter is divided into four types: transporters, carriers, enzymes, and targets. All four types are combined and arranged into a protein layer, which will be connected to the drug layer. The type is added as an attribute to the nodes.

After calling loadDBXML(), the functions getDBDrug() and getDBInteractions() can be called to fetch respectively the drug entries’ information and the interactions between them from DrugBank, using the parsed DrugBank object, and the list of DrugBank IDs. The addition of a Drug Layer to an existing mully graph can be achieved using the function addDBLayer(), with the mully graph, the parsed DrugBank object, and the list of DrugBank IDs as arguments.

The connections to the protein nodes on the protein Layer are returned by the function getDBtoUPKB(), which combines the results of the four following functions: getDBTargets(), getDBTransporters(), getDBCarriers(), and getDBEnzymes().

#### 3.1.2. UniProt

**Figure 3:**
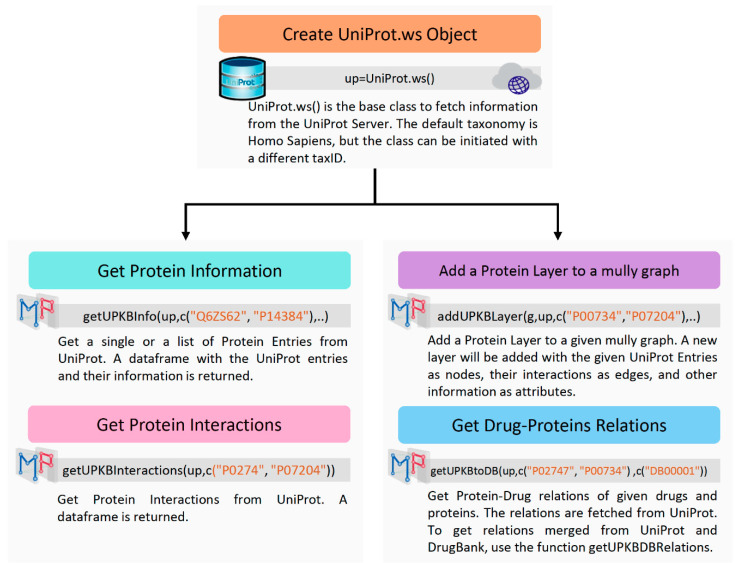
UniProt Workflow Diagram. The Diagram shows the steps to fetch information on Proteins from UniProt using Multipath and the R Package UniProt.ws.

The Multipath package uses the R interface UniProt.ws [6] to query the UniProt database. UniProt.ws contains functions for retrieving, processing and repackaging the UniProt web services. We use the package to get information on proteins, their interactions, and their connections to DrugBank. The information extracted from UniProt is used to add a Protein Layer to a mully graph, with the interactions between the proteins, and the connections to the drugs on the Drug Layer, if contained in the graph. All functions related to UniProt require the UniProt.ws object obtained by calling UniProt.ws() (Figure 3).

To add the protein layer, the information on a list of proteins can be extracted using addUPKBInfo(), with the UniProt.ws object, the list of UniProt IDs and the columns’ names representing the attributes as an input. To get the list of possible columns, the UniProt.ws function columns() can be called. To get the edges between the protein nodes, the function getUPKBInteractions() can be called, returning the list of edges, needed alongside the list of nodes to build the protein layer. This task can be accomplished using the function addUPKBLayer(), with the mully graph, the UniProt.ws object, the list of UniProt IDs, and the list of attributes as arguments.

The connections to the Drug Layer can be obtained by calling the function getUPKBtoDB().

The addition of the Protein and Drug Layers can also be automatically achieved by calling the function multipath(), with the following arguments: the name of the returned mully graph, the UniProt.ws object, the list of UniProt IDs, the DrugBank parsed object, and the list of DrugBank IDs.

### 3.2. Pathway Models’ Reproducibility

The main feature of Multipath is the possibility to track modifications applied to the multilayered graph built from the pathway BioPAX file. The user can apply a set of standard procedures, i.e., the addition or removal of nodes, edges, and layers. Every modification is stored in a pathwayView object, which can be used later to undo these modifications, retrace the transformation of the original graph, apply further modifications or compare to other views generated from the same pathway.

## 4. Discussion

### 4.1. Wnt Multipath Model

**Figure 4:**
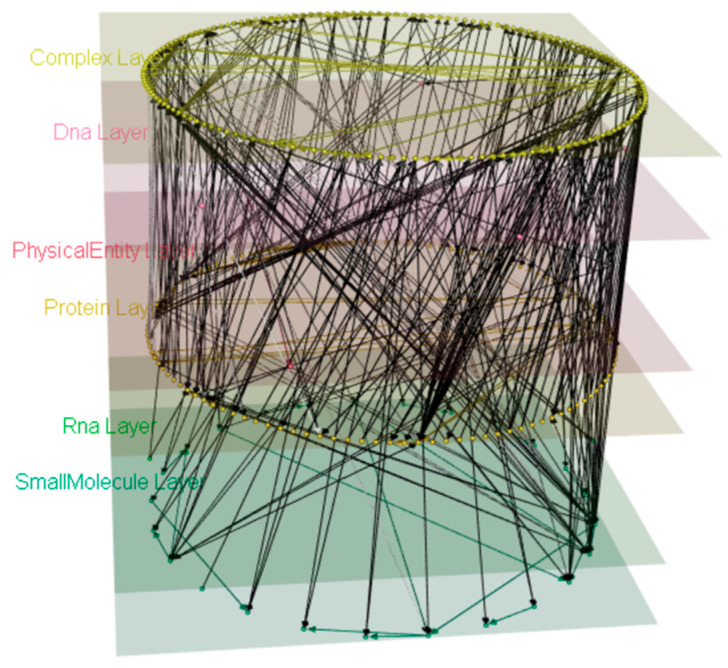
Signaling by Wnt mully model. The model was built using the BioPAX level 3 Signaling by Wnt from Reactome. The model has 6 layers, 311 nodes, and 539 edges.

The Wnt signaling pathway is an ancient and evolutionarily conserved pathway that regulates crucial aspects of cell fate determination, cell migration, cell polarity, neural patterning, and organogenesis during embryonic development [17]. We generated a multilayered model (Figure 4) of the Signaling by Wnt pathway (BioPAX level 3) [18] from the Reactome Database [9].

**Figure 5:**
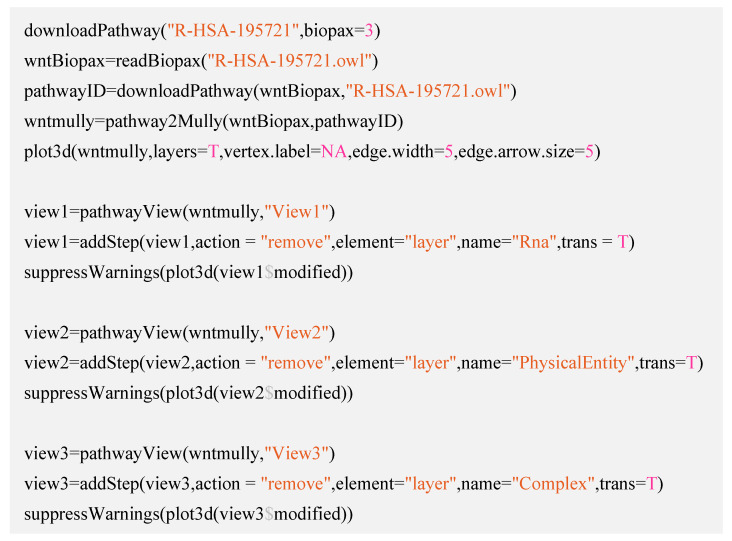
The script to generate and plot the model and the views. The function pathway2Mully() transforms the pathways parsed in the Biopax object into mully graphs. To plot the graph, we used the function plot3d() from the mully package, and finally, we generated the views using pathwayView() and modified them using the functions addStep() which applies a modification step to the graph.

To build the model and generate the views, we used the R Script in Figure 5.

The downloaded pathway file (4.19 MB) was parsed in 24.56 s and occupies ~5.05 MB of memory space. The transformation to a mully graph was conducted in 19.23 s, and the resulted graph’s size is 121.744 KB.

**Figure 6:**
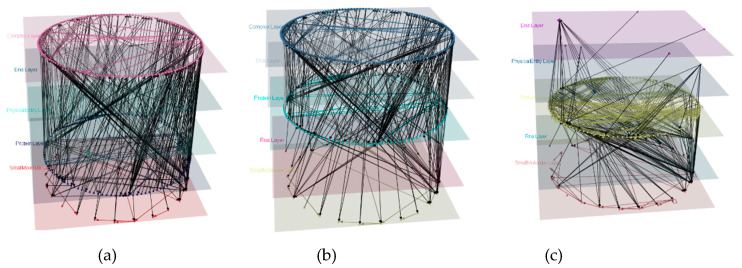
Views generated from the Signaling by Wnt mully model. (**a**) View1 generated by deleting the RNA layer. The graph has 5 layers, 309 nodes, and 533 edges. (**b**) View2 generated by deleting the PhysicalEntity layer. The graph has 5 layers, 308 nodes, and 522 edges (521 edges from the original graph and 1 transitive). (**c**) View3 generated by deleting the Complex layer. The graph has 5 layers, 153 nodes and 800 edges (93 edges from the original graph and 707 transitive).

We applied different changes to the model and generated three different views from the model (Figure 6), by deleting the RNA (Figure 6a), Physical Entity (Figure 6b), and Complex (Figure 6c) layers respectively. Upon deletion of the layers, transitive edges derived from the deleted nodes were added. All deleted information can be reversed from the view. The original model (Figure 4) can be reproduced from either of these three views using the function undo.

### 4.2. Adding a Drug Layer

**Figure 7:**
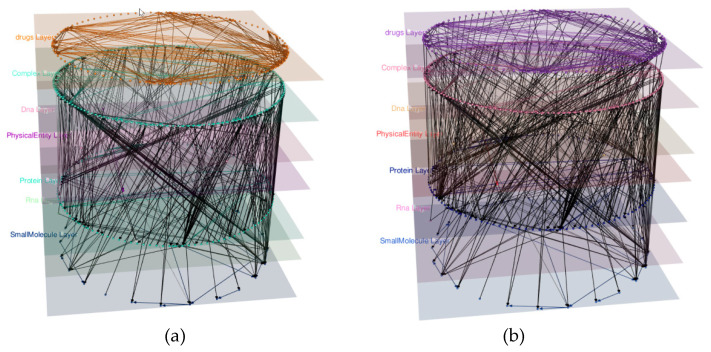
The Signaling by Wnt model with the added Drug Layer. (**a**) The model after adding the Drug layer. The layer contains 83 drugs and 260 Drug Interactions. (**b**) The model after adding the connections between the drugs and proteins’ nodes, merged from DrugBank and UniProt. The Drug targets obtained are 97, many of them added as multi-edges, since a single internal protein ID was mapped to multiple UniProt entries.

After creating the Signaling by Wnt Pathway model, we retrieved the Drugs targeting the proteins on the protein layer. We extracted the mappings of the internal IDs to UniProt using the function getExternalIDs(). We obtained the list of Drugs targeting these proteins using getUPKBtoDB(). These IDs were used to add a Drug layer to our model using addDBLayer(), which automatically adds the Drugs and their interactions to the graph. To get the merged Drug–Protein targets from both databases, we used the function getUPKBDBRelations() using the list of external Protein IDs and the list of DrugBank IDs. The edges were added then one by one (Figure 7).

To retrieve drug information from DrugBank, parsing the downloaded data from DrugBank is required, which is a relatively slow process, fortunately needed only once before calling any drug-related functions. The parsed data is a large list occupying ~553MB of memory space. However, extracting the drug information and interactions, as well as their targets is fast. On the other hand, the retrieval of protein information is accomplished by means of the UniProt.ws R Package. This process does not require any memory space, but we faced a very slow response from the UniProt server multiple times, with no detection of response speed patterns, i.e. specific time of the day, or the amount of information to be retrieved. The web service also recommends querying using a maximum number of 50 UniProt entries’ IDs in each single query. Multipath could be updated in the future to support a better API/Tool for this process. 

## 5. Conclusions

Multipath is an R package to generate multilayered models from BioPAX encoded pathway knowledge. The models are modifiable, and all modifications are tracked to allow the reproduction of the graphs. Multipath can also be used to query influencing knowledge databases such as UniProt and DrugBank and integrate them into a multilayered graph. In this paper, we presented the different features of Multipath. We described how to use our package to generate multilayered models from BioPAX files, and integrate different pathway knowledge. We used the Signaling by Wnt pathway as a demonstration, and displayed three different views derived from it. Multipath is open source, and can be downloaded from our GitHub Repository [19]. The reference manual and the vignette of the package were added as Supplementary Materials, respectively as Files S1 and S2.

## Supplementary Materials

The following are available online at https://www.mdpi.com/2079-7737/9/12/483/s1, File S1: The package’s vignette in the PDF format, File S2: The package’s Reference Manual.
