# Supplementary material for: Multipath: An R Package to Generate Integrated Reproducible Pathway Models
Source: Biology (Basel). 2020 Dec 21;9(12):483. doi: 10.3390/biology9120483 (PMC7766416; doi:10.3390/biology9120483)
Supplement: Supplementary file 1 [file biology-09-00483-s001.zip › biology-993658-Supplementary Materials/File S1.pdf]

Multipath - an R package to create integrated reproducible multilayered pathway models

true

2020-11-04

Introduction

Biological pathway data integration has become a topic of interest in the past years. This interest originates essentially from the continuously increasing size of existing prior knowledge as well as from the many challenges scientists face when studying biological pathways. Multipath is a framework that aims at helping re-trace the use of specific pathway knowledge in specific publications, and easing the data integration of multiple pathway types and further influencing knowledge sources. Using Multipath, BioPax-encoded pathways can be parsed and embedded into multilayered graphs. Modifications can be applied to these graphs to generate different views. The package is implemented as a part of the Multipath Project directed by Dr. Frank Kramer .

Installation

Preinstallation

Multipath depends on multiple packages. The packages are the following: UniProt.ws, dbparser, rBiopaxParser, mully, TCGAretriever, stringr, stringi, svMisc, uuid, dplyr, crayon Please make sure to install the packages UniProt.ws,rBiopaxParser and mully before using the package.

To install the UniProt.ws package:

```
if (!requireNamespace("BiocManager", quietly = TRUE))
    install.packages("BiocManager")

BiocManager::install("UniProt.ws")
```

To install the mully package:

```
require(devtools)
install_github("frankkramer-lab/mully")
library(mully)
```

To install the rBiopaxParser package:

```
require(devtools)
install_github("frankkramer-lab/rBiopaxParser")
library(rBiopaxParser)
```

Installation via Github

```
require(devtools)
install_github("frankkramer-lab/Multipath")
library(Multipath)
```

Available Functions

addDBLayer

Add a drug layer to a mully graph

This function is used to add a DrugBank layer to an existing mully model. The function needs the following arguments:

- **g** - The mully graph
- **drugList** - The list of DrugBank Ids of the drugs to be added. This argument can be either a string (one drug) or a list of strings (multiple drugs)
- **data** - The dataframe containing the parsed information of DrugBank. This argument can be obtained using the function `loadDBXML(DrugBankFile)`

The function returns a mully graph with the added drug layer

Example

```
g=mully("DrugBank",direct=T)
data=loadDBXML("DrugBank.xml")
g=addDBLayer(g,data,c("DB00001","DB06605"))
```

addStep

Track a modification of a graph

The function saves a modification applied to a mully graph. It applies the step to the graph and saves the modification step in the pathwayView Object. Not all of the arguments are mandatory, they depend on the step that has to be applied. The function needs the following arguments:

- **v** - The input view in which the modification should be saved
- **action** - The type of action to be applied. Can either be “add” or "remove
- **element** - The type of the element to be modified. Can either be “node”, “edge”, or “layer”
- **name** - The name of the element to be modified. This argument is only mandatory for nodes and edges
- **layername** - The layer name. This argument is only mandatory for action “add” and element “node”
- **V1** - The start node of an edge. This argument is only mandatory for element “edge”
- **V2** - The end node of an edge. This argument is only mandatory for element “edge”
- **attributes** - The named list of attributes of the element. This argument is required only for action “add”. It is optional for both elements “node” and “edge”, but mandatory if the edge alread exists
- **multi** - A boolean whether to select multi-edges or not. This is only mandatory for action “remove” and element “edge”. By default set to FALSE, in which case the attributes of the specified edge should be given

- **trans** - A boolean whether to add transitive edges upon removal of nodes or layers

The function returns the view with the added step. ***Example***

```
g=mully:::demo()
view=pathwayView(g,"View1")
view=addStep(view,"remove","layer","disease")
```

addUPKBLayer

Add a protein layer to a mully graph

This function is used to add a UniProt protein layer to an existing mully model. The function needs the following arguments: - **g** - The mully graph - **up** - The UniProt.ws Object - **proteinList** - The list of UniProt Ids of the proteins to be added - **col** - The list of attributes associated to the UniProtKB Entries to be retrieved

The function returns the mully graph with the added UniProt layer The function should be preceded by `UniProt.ws()` to get the UniProt.ws Object

Example

```
up=UniProt.ws()
g=mully("UniProt")
g=addUPKBLayer(g,up,proteinList=c("P02747","P00734","P07204"),col=c("UNIPROTKB","PROTEIN-NAMES"))
```

downloadPathway

Download Reactome Pathways in BioPAX level 2 and 3

This function is used to download one or a list of pathways, encoded in BioPAX level 2 or 3. The function needs the following arguments:

- **pathwayID** - The Reactome ID or list of IDs of the pathways to be downloaded. The ID should start with R-HSA-.
- **biopaxLevel** - The BioPAX Level, 2 or 3. By default set to 3.
- **destDirectory** - The Directory in which the Pathway Files should be saved. If missing, the files are saved in the working directory. The Reactome IDs are used to name the files.
- **overwrite** - A Boolean whether to overwrite existing files with the same name.

The function returns the path to the directory in which the files are downloaded.

Example

```
downloadPathway(c("R-HSA-195721","R-HSA-9609507"),biopaxLevel=3,overwrite=T)
```

getAllUPKB

Get all proteins' entries from UniProt

The function is used to fetch all protein entries from UniProt. The function needs the following arguments: - **up** - The UniProt.ws Object

The function returns a dataframe containing the Protein's entries with the ID and Name.

Should be preceded by `UniProt.ws()` to get the UniProt.ws Object

Example

```
up=UniProt.ws()
allProteins=getAllUPKB(up)
```

getDBCarriers

Get the Carriers Protein Targets of given DrugBank drugs

Protein Targeted by Drugs are divided in DrugBank into 4 types: Targets, Enzymes, Carriers and Transporters. This function is used to extract the carriers from the dataframe containing the information on the drugs parsed from the DrugBank XML File.

The function needs the following arguments:

- **data** - The dataframe containing the parsed information of DrugBank. This argument can be obtained using the function `loadDBXML(DrugBankFile)`
- **drugList** - The list of DrugBank Ids of the drugs. This argument can be either a string (one drug) or a list of strings (multiple drugs)

The function returns a dataframe containing all information on the carriers targeted by the given drug list.

Example

```
data=loadDBXML(DrugBankFilePath)
getDBCarriers(data,"DB00001")
```

getDBDrug

Get DrugBank drug entry

This function extracts infromation on one or a list of Drugs from the dataframe parsed from the DrugBank XML file. The function needs the following arguments:

- **data** - The dataframe containing the parsed information of DrugBank. This argument can be obtained using the function `loadDBXML(DrugBankFile)`
- **drug** - The ID or list of IDs of the DrugBank drug entries starting with "DB"

This function returns a dataframe containing the DrugBank entry with its information

Example

```
data=loadDBXML(DrugBankFilePath)
getDBDrug(data,"DB00001")
```

getDBDrugInteractions

Get DrugBank Drug to Drug Interactions This function is used to extract Drug Interactions from the dataframe containing the information on the Drug in DrugBank, parsed from the downloaded XML File.

The function needs the following arguments:

- **data** - The dataframe containing the parsed information of DrugBank. This argument can be obtained using the function `loadDBXML(DrugBankFile)`
- **drug** - The ID of the DrugBank drug entry starting with "DB". This argument can be either a string (one drug) or a list of strings (multiple drugs).

The function returns a dataframe containing the DrugBank interactions in which the given drug is involved
Example

```
data=loadDBXML("DrugBank.xml")
getDBDrugInteractions(data,"DB06605")
```

getDBEnzymes

Get the Enzyme Protein Targets of given DrugBank drugs

Protein Targeted by Drugs are divided in DrugBank into 4 types: Targets, Enzymes, Carriers and Transporters. This function is used to extract the enzymes from the dataframe containing the information on the drugs parsed from the DrugBank XML File.

The function needs the following arguments:

- **data** - The dataframe containing the parsed information of DrugBank. This argument can be obtained using the function `loadDBXML(DrugBankFile)`
- **drugList** - The list of DrugBank Ids of the drugs. This argument can be either a string (one drug) or a list of strings (multiple drugs)

The function returns a dataframe containing all information on the enzymes targeted by the given drug list.

Example

```
data=loadDBXML(DrugBankFilePath)
getDBEnzymes(data,"DB00001")
```

getDBTargets

Get the Target Protein Targets of given DrugBank drugs

Protein Targeted by Drugs are divided in DrugBank into 4 types: Targets, Enzymes, Carriers and Transporters. This function is used to extract the targets from the dataframe containing the information on the drugs parsed from the DrugBank XML File.

The function needs the following arguments:

- **data** - The dataframe containing the parsed information of DrugBank. This argument can be obtained using the function `loadDBXML(DrugBankFile)`
- **drugList** - The list of DrugBank Ids of the drugs. This argument can be either a string (one drug) or a list of strings (multiple drugs)

The function returns a dataframe containing all information on the targets of the given drug list.

Example

```
data=loadDBXML(DrugBankFilePath)
getDBTargets(data,"DB00001")
```

getDBTransporters

Get the Transporters Protein Targets of given DrugBank drugs

Protein Targeted by Drugs are divided in DrugBank into 4 types: Targets, Enzymes, Carriers and Transporters. This function is used to extract the transporters from the dataframe containing the information on the drugs parsed from the DrugBank XML File.

The function needs the following arguments:

- **data** - The dataframe containing the parsed information of DrugBank. This argument can be obtained using the function `loadDBXML(DrugBankFile)`
- **drugList** - The list of DrugBank Ids of the drugs. This argument can be either a string (one drug) or a list of strings (multiple drugs)

The function returns a dataframe containing all information on the transporters targeted by the given drug list.

Example

```
data=loadDBXML(DrugBankFilePath)
getDBTransporters(data,"DB00001")
```

getDBtoUPKB

Get DrugBank Drugs to UniProt Proteins Relations from DrugBank

This function is used to extract Drug Targets from the dataframe containing the information on the drugs parsed from the DrugBank XML File. It merges the targets returned by 4 functions: enzymes, targets, transporters and carriers. The function needs the following arguments:

- **data** - The dataframe containing the parsed information of DrugBank. This argument can be obtained using the function `loadDBXML(DrugBankFile)`
- **drugList** - The list of DrugBank Ids of the drugs. This argument can be either a string (one drug) or a list of strings (multiple drugs)
- **proteinList** - The list of UniProt Ids of the proteins

The function returns a dataframe containing the connections between DrugBank drugs and UniProt proteins retrieved from DrugBank.

Example

```
data=loadDBXML("DrugBank.xml")
getDBtoUPKB(data,c("DB00001","DB00002","DB00006"),c("P02747","P00734","P07204","P05164"))
```

getPathwayID

Get internal pathway ID in a BioPAX file

This function is used to get the internal ID of a pathway in a parsed BioPAX object. A BioPAX file can contain multiple pathways, indexed internally using ID starting with "Pathway" followed by the number of the pathway. Each pathway in the file has a Reactome and an internal ID. The latter can be extracted using this function. This should be preceded by `readBiopax(filepath)` to obtain the biopax object The function needs the following arguments:

- **biopax** - The biopax object
- **reactomeID** - The Reactome ID of the pathway

The function returns the internal ID of the pathway in the parsed BioPAX object.

Example

```
biopax=readBiopax("pi3k.owl")
id=getPathwayID(biopax,"R-HSA-167057")
pi3kmully=pathway2mully(biopax,id)
```

getUPKBDBRelations

Get Protein and Drugs relations from UniProt and DrugBank

The function is used to obtain drug targets from UniProt and DrugBank. It combines the returned relations from both functions `getDBtoUPKB` and `getUPKBtoDB`. The function needs the following arguments:

- **up** - The UniProt.ws Object
- **data** - The dataframe containing the parsed information of DrugBank. This argument can be obtained using the function `loadDBXML(DrugBankFile)` -**proteinList** - The list of UniProt Ids of the proteins
- **drugList** - The list of DrugBank Ids of the drugs. This argument can be either a string (one drug) or a list of strings (multiple drugs)

The function returns a dataframe containing the connections between DrugBank drugs and UniProt proteins retrieved from DrugBank and UniProt. The function should be preceded by:

1. `UniProt.ws()` to get the UniProt.ws Object
2. `loadDBXML(DrugBankFile)` to get the argument data

Example

```
up=UniProt.ws()
data=loadDBXML("DrugBank.xml")
relations=getUPKBDBRelations(up,data,proteinList=c("P02747","P00734","P07204","P05164"),drugList=c("DB0
```

getUPKBInfo

Get Proteins from UniProtKB

The function is used to fetch information on a list of protein entries from UniProt. The function needs the following arguments: - **up** - The UniProt.ws Object - **proteins** - The list of UniProtKB Proteins ID to be retrieved - **col** - The list of attributes associated to the UniProtKB Entries to be retrieved

The function returns a dataframe containing the protein entries with the selected attributes. To get the list of possible columns, you can call `columns(UniProt.ws())`. The function should be preceded by `UniProt.ws()` to get the UniProt.ws Object.

Example

```
up <- UniProt.ws()
getUPKBInfo(up,c("Q6ZS62","P14384","P40259"),c("PROTEIN-NAMES","DRUGBANK","GO","REACTOME"))
```

getUPKBInteractions

Get the interactions of given proteins from UniProt

The function is used to fetch interactions between proteins from the UniProt Database. The function needs the following arguments:

- **up** - The UniProt.ws Object
- **proteins** - The list of proteins of which the interactions should be retrieved

The function returns a dataframe containing the interactions between the given proteins. The function should be preceded by `UniProt.ws()` to get the UniProt.ws Object.

Example

```
up=UniProt.ws()
interactions=getUPKBInteractions(up,c("P02747","P07204","P00734"))
```

getUPKBtoDB

Get UniProt Proteins to DrugBank Drugs relations from UniProt

This function is used to fetch relations between a list of proteins and a list of drugs from the UniProt Database. The function needs the following arguments:

- **up** - The UniProt.ws Object
- **proteinList** - The list of UniProt Ids of the proteins
- **drugList** - The ID of the DrugBank drug entry starting with "DB". This argument can be either a string (one drug) or a list of strings (multiple drugs).

The function returns a dataframe containing the connections between UniProt proteins and DrugBank drugs retrieved from UniProt. The function should be preceded by `UniProt.ws()` to get the UniProt.ws Object.

Example

```
up=UniProt.ws()
getUPKBtoDB(up,c("P02747","P00734","P07204"),c("DB00001","DB00002"))
```

loadDBXML

Load DrugBank XML file

This function is used to read and parse the file downloaded from the DrugBank Database containing the complete information on the drug entries. The function needs the following argument:

- **file** - The path to the DrugBank XML file. This can be downloaded from the DrugBank official Website (drugbank.ca). An account with an institutional e-mail is required.

This function returns a dataframe containing the parsed information from DrugBank. This can be used to extract any additional information on the DrugBank entries

This function should be called before using any function to query the DrugBank database. Since the parsing of DrugBank takes time, this function should only be called once.

Example

```
data=loadDBXML("DrugBank.xml")
```

multipath

Generate Multipath Graph from General Data

This function is used to generate a mully graph from a list of drugs and proteins. The function creates a multilayered graph with a drug and protein layer, and adds the inter- and intractions to it. The function needs the following arguments:

- **name** - The name of the graph to be generated
- **up** - The `Uniprot.ws()` object
- **proteinList** - The list of proteins of which the interactions should be retrieved
- **data** - The dataframe containing the parsed information of DrugBank. This argument can be obtained using the function `loadDBXML(DrugBankFile)`
- **drugList** - The list of DrugBank Ids of the drugs. This argument can be either a string (one drug) or a list of strings (multiple drugs)

The function returns a mully graph with the added data. The function should be preceded by:

1. `UniProt.ws()` to get the UniProt.ws Object
2. `loadDBXML(DrugBankFile)` to get the argument data

Example

```
up=UniProt.ws()
data=loadDBXML("DrugBank.xml")
g=multipath(name="MyMultipath",up=up,proteinList=c("P02747","P00734","P07204","P05164"),data=data,drugL
```

pathway2Mully

Build a mully graph from a given pathway This function builds a multilayered mully graph of a BioPAX encoded pathway. To run this function, the user needs to parse the file. It should be preceded by `readBiopax(filepath)` to obtain the biopax object. The function needs the following arguments:

- **biopax** - The BioPaX object containing the parsed data from an OWL file. This can be obtained using `readBiopax(filepath)`
- **pathwayID** - The internal ID of the pathway in the biopax object. To obtain the internal ID, the function `getPathwayID(biopax,reactomeID)` can be called

The function returns a mully graph built from the given pathway.

Example

```
biopax=readBiopax("pi3k.owl")
pi3kmully=pathway2mully(biopax,"pathway1")
```

pathwayView

Create an empty view

The function is used to create a pathwayView in order to track the modifications applied to a mully graph. The ocject pathwayView contains different information on the View, including the timestamp of creation and last modification, the original and final version of the graph, and the dataframe containing the modification steps. The function needs the following arguments:

- **g** - The input graph
- **name** - The name of the view

The function returns an empty pathwayView Object.

Example

```
view=pathwayView(mully("myMully",T),"View1")
```

print,pathwayView

Print Function

The function is used to print the pathwayView Object. The function needs the following arguments:

- **v** - The input pathwayView to be printed

undo

Undo a modification step in a view

The function reverses changes applied to a mully graph, saved in a pathwayView Object. The function needs the following arguments:

- **v** - The input view
- **stps** - The number of steps to undo. This number referes to the number of unique steps' IDs to be removed, i.e. entries of steps in the view with similar stepID count as 1

The function returns The view with the undone modifications. ## wntpathway **Demo function for Wnt Pathway Views** The function is a demo function that create a pathway mully graph from a BioPAX encoded file of the Signaling by Wnt Pathway. The function reads and parses the file, creates the mully graph, and generates 3 different views from the graph by deleting the RNA, Complex, and Physical Entity Layers. The function needs the following arguments:

- **file** - The link to the Wnt Pathway bioPAX file

Example

```r
wntpathway("wnt_reactome.owl")
```
