# Supplementary material for: Multipath: An R Package to Generate Integrated Reproducible Pathway Models
Source: Biology (Basel). 2020 Dec 21;9(12):483. doi: 10.3390/biology9120483 (PMC7766416; doi:10.3390/biology9120483)
Supplement: Supplementary file 1 [file biology-09-00483-s001.zip › biology-993658-Supplementary Materials/File S2.pdf]

Package ‘Multipath’

December 10, 2020

Type Package

Title Pathway Data Integration With Influencing Knowledge Using Multi-Layered Graphs

Version 1.0.3

Author Zaynab Hammoud

Maintainer Zaynab Hammoud <zaynabhassanhammoud@gmail.com>

Description Allows the integration of pathway data with influencing knowledge from different databases, like DrugBank, Reactome and UniProt, by using multilayered graphs.

License GPL (¿= 2)

Encoding UTF-8

LazyData true

Depends stringr

RoxygenNote 7.1.1

Suggests knitr,
rmarkdown

Imports mully (¿= 2.1.26),
UniProt.ws,
dbparser,
rBiopaxParser,
TCGAretriever,
svMisc,
uuid,
dplyr,
crayon,
igraph,
RCurl,
graph

VignetteBuilder knitr

R topics documented:

addDBLayer 2
addStep 3
addUPKBLayer 4
downloadPathway 5
getAllUPKB 5

getDBCarriers . . . 6
getDBDrug . . . 6
getDBDrugInteractions . . . 7
getDBEnzymes . . . 8
getDBTargets . . . 8
getDBtoUPKB . . . 9
getDBTransporters . . . 9
getExternalIDs . . . 10
getPathwayID . . . 10
getUPKBDBRelations . . . 11
getUPKBInfo . . . 12
getUPKBInteractions . . . 12
getUPKBtoDB . . . 13
loadDBXML . . . 14
multipath . . . 14
pathway2Mully . . . 15
pathwayView . . . 16
print.pathwayView . . . 16
undo . . . 17
wntpathway . . . 17

Index 18

`addDBLayer` *Add a drug layer to a mully graph*

Description

Add a drug layer to a mully graph

Usage

```
addDBLayer(g, data, drugList)
```

Arguments

`g`	The mully graph
`data`	The dataframe containing the parsed information of DrugBank. This argument can be obtained using the function loadDBXML(DrugBankFile)
`drugList`	The list of DrugBank Ids of the drugs to be added. This argument can be either a string (one drug) or a list of strings (multiple drugs)

Value

A mully graph with the added drug layer

Examples

```
## Not run:
data=readDBXML(DBXMLFilePath)
g=mully("DrugBank",direct=T)
g=addDBLayer(g,data,c("DB00001","DB06605"))

## End(Not run)
```

addStep *Track a modification of a graph*

Description

Track a modification of a graph

Usage

```
addStep(
  v,
  action,
  element,
  name = NA,
  layername = NA,
  V1 = NA,
  V2 = NA,
  attributes = NA,
  multi = F,
  trans = T
)
```

Arguments

v	The input view in which the modification should be saved
action	The type of action to be applied. Can either be "add" or "remove
element	The type of the element to be modified. Can either be "node", "edge", or "layer"
name	The name of the element to be modified. This argument is only mandatory for nodes and edges
layername	The layer name. This argument is only mandatory for action "add" and element "node"
V1	The start node of an edge. This argument is only mandatory for element "edge"
V2	The end node of an edge. This argument is only mandatory for element "edge"
attributes	The named list of attributes of the element. This argument is required only for action "add". It is optional for both elements "node" and "edge", but mandatory if the edge alread exists
multi	A boolean whether to select multi-edges or not. This is only mandatory for action "remove" and element "edge". By default set to FALSE, in which case the attributes of the specified edge should be given
trans	A boolean whether to add transitive edges upon removal of nodes or layers

Value

The View with the added step

Examples

```
## Not run:
g=mully:::demo()
view=pathwayView(g,"View1")
view=addStep(view,"remove","layer","")

## End(Not run)
```

---

addUPKBLayer *Add a protein layer to a mully graph*

Description

Add a protein layer to a mully graph

Usage

```
addUPKBLayer(
  g,
  up,
  proteinList,
  col = c("UNIPROTKB", "PROTEIN-NAMES", "ORGANISM")
)
```

Arguments

g	The mully graph
up	The UniProt.ws Object
proteinList	The list of UniProt Ids of the proteins to be added
col	The list of attributes associated to the UniProtKB Entries to be retrieved

Value

The mully graph with the added UniProt layer

Note

Should be preceded by UniProt.ws() to get the UniProt.ws Object

Examples

```
## Not run:
up=UniProt.ws()
g=mully("UniProt")
g=addUPKBLayer(g,up,proteinList=c("P02747","P00734","P07204"),col=c("UNIPROTKB","PROTEIN-NAMES"))

## End(Not run)
```

`downloadPathway` *Download Reactome Pathways in BioPAX level 2 and 3*

Description

Download Reactome Pathways in BioPAX level 2 and 3

Usage

```
downloadPathway(pathwayID, biopaxLevel = "3", destDirectory, overwrite = F)
```

Arguments

`pathwayID`	The Reactome ID or list of IDs of the pathways to be downloaded. The ID should start with R-HSA-.
`biopaxLevel`	The BioPAX Level, 2 or 3. By default set to 3.
`destDirectory`	The Directory in which the Pathway Files should be saved. If missing, the files are saved in the working directory. The Reactome IDs are used to name the files.
`overwrite`	A Boolean whether to overwrite existing files with the same name.

Value

The Directory in which the files are saved.

Examples

```
## Not run:
downloadPathway(c("R-HSA-195721","R-HSA-9609507"),biopaxLevel=3,overwrite=T)

## End(Not run)
```

`getAllUPKB` *Get all proteins' entries from UniProt*

Description

Get all proteins' entries from UniProt

Usage

```
getAllUPKB(up)
```

Arguments

`up`	The UniProt.ws Object

Value

a dataframe containing the Protein's entries with the ID and Name

Note

Should be preceded by UniProt.ws() to get the UniProt.ws Object

Examples

```
## Not run:
up=UniProt.ws()
allProteins=getAllUPKB(up)

## End(Not run)
```

`getDBCarriers` *Get the carrier proteins involved in movement of given drugs across biological membranes*

Description

Get the carrier proteins involved in movement of given drugs across biological membranes

Usage

```
getDBCarriers(data, drugList)
```

Arguments

`data`	The dataframe containing the parsed information of DrugBank. This argument can be obtained using the function loadDBXML(DrugBankFile)
`drugList`	The list of DrugBank Ids of the drugs. This argument can be either a string (one drug) or a list of strings (multiple drugs)

Value

A dataframe containing all information on the carrier proteins involved in movement of the given drugs across biological membranes

---

`getDBDrug` *Get DrugBank drug entry*

Description

Get DrugBank drug entry

Usage

```
getDBDrug(data, drug)
```

Arguments

`data`	The dataframe containing the parsed information of DrugBank. This argument can be obtained using the function loadDBXML(DrugBankFile)
`drug`	The ID or list of IDs of the DrugBank drug entries starting with "DB"

Value

A dataframe containing the DrugBank entry with its information

Examples

```
## Not run:
data=loadDBXML(DrugBankFilePath)
getDBDrug(data, "DB00001")

## End(Not run)
```

---

getDBDrugInteractions *Get DrugBank Drug to Drug Interactions*

Description

Get DrugBank Drug to Drug Interactions

Usage

```
getDBDrugInteractions(data, drug)
```

Arguments

data	The dataframe containing the parsed information of DrugBank. This argument can be obtained using the function loadDBXML(DrugBankFile)
drug	The ID of the DrugBank drug entry starting with "DB". This argument can be either a string (one drug) or a list of strings (multiple drugs).

Value

A dataframe containing the DrugBank interactions in which the given drug is involved

Examples

```
## Not run:
data=loadDBXML(DBXMLFilePath)
getDBDrugInteractions(data,"DB06605")

## End(Not run)
```

`getDBEnzymes` *Get the enzymes inhibited/induced or involved in metabolism by given DrugBank drugs*

Description

Get the enzymes inhibited/induced or involved in metabolism by given DrugBank drugs

Usage

```
getDBEnzymes(data, drugList)
```

Arguments

`data`	The dataframe containing the parsed information of DrugBank. This argument can be obtained using the function loadDBXML(DrugBankFile)
`drugList`	The list of DrugBank Ids of the drugs. This argument can be either a string (one drug) or a list of strings (multiple drugs)

Value

A dataframe containing all information on the enzymes inhibited/induced or involved in metabolism by the given drug list

`getDBTargets` *Get the targets of given DrugBank drugs*

Description

Get the targets of given DrugBank drugs

Usage

```
getDBTargets(data, drugList)
```

Arguments

`data`	The dataframe containing the parsed information of DrugBank. This argument can be obtained using the function loadDBXML(DrugBankFile)
`drugList`	The list of DrugBank Ids of the drugs. This argument can be either a string (one drug) or a list of strings (multiple drugs)

Value

A dataframe containing all information on the targets of the given drug list

`getDBtoUPKB` *Get DrugBank Drugs to UniProt Proteins Relations from DrugBank*

Description

Get DrugBank Drugs to UniProt Proteins Relations from DrugBank

Usage

```
getDBtoUPKB(data, drugList, proteinList)
```

Arguments

`data`	The dataframe containing the parsed information of DrugBank. This argument can be obtained using the function loadDBXML(DrugBankFile)
`drugList`	The list of DrugBank Ids of the drugs. This argument can be either a string (one drug) or a list of strings (multiple drugs)
`proteinList`	The list of UniProt Ids of the proteins

Value

A dataframe containing the connections between DrugBank drugs and UniProt proteins retrieved from DrugBank

Examples

```
## Not run:
data=readDBXML(DBXMLFilePath)
getDBtoUPKB(data,c("DB00001","DB00002","DB00006"),c("P02747","P00734","P07204","P05164"))

## End(Not run)
```

`getDBTransporters` *Get the transporter proteins involved in movement of given drugs across biological membranes*

Description

Get the transporter proteins involved in movement of given drugs across biological membranes

Usage

```
getDBTransporters(data, drugList)
```

Arguments

`data`	The dataframe containing the parsed information of DrugBank. This argument can be obtained using the function loadDBXML(DrugBankFile)
`drugList`	The list of DrugBank Ids of the drugs. This argument can be either a string (one drug) or a list of strings (multiple drugs)

Value

A dataframe containing all information on the transporter proteins involved in movement of the given drugs across biological membranes

---

getExternalIDs — *Get External Database IDs of nodes*

Description

Get External Database IDs of nodes

Usage

```
getExternalIDs(biopax, nodes, database)
```

Arguments

`biopax`	The biopax object
`nodes`	The list of internal IDs of the nodes
`database`	The name of the database

Value

A dataframe with the mappings between the internal and external IDs

Examples

```
## Not run:
biopax=readBiopax(pi3k.owl)
getExternalIDs(wntBiopax,c("Protein1","Protein2"),"UniProt")

## End(Not run)
```

---

getPathwayID — *Get internal pathway ID in a BioPAX file*

Description

Get internal pathway ID in a BioPAX file

Usage

```
getPathwayID(biopax, reactomeID)
```

Arguments

`biopax`	The biopax object
`reactomeID`	The Reactome ID of the pathway

Value

The internal ID of the pathway in the parsed BioPAX object

Note

This should be preceded by readBiopax(filepath) to obtain the biopax object

Examples

```
## Not run:
biopax=readBiopax(pi3k.owl)
id=getPathwayID(biopax,"R-HSA-167057")
pi3kmully=pathway2mully(biopax,id)

## End(Not run)
```

---

getUPKBDBRelations *Get Protein and Drugs relations from UniProt and DrugBank*

Description

Get Protein and Drugs relations from UniProt and DrugBank

Usage

```
getUPKBDBRelations(up, data, proteinList, drugList)
```

Arguments

`up`	The UniProt.ws Object
`data`	The dataframe containing the parsed information of DrugBank. This argument can be obtained using the function loadDBXML(DrugBankFile)
`proteinList`	The list of UniProt Ids of the proteins
`drugList`	The list of DrugBank Ids of the drugs. This argument can be either a string (one drug) or a list of strings (multiple drugs)

Value

A dataframe containing the connections between DrugBank drugs and UniProt proteins retrieved from DrugBank and UniProt

Note

Should be preceded by: 1. UniProt.ws() to get the UniProt.ws Object 2. loadDBXML(DrugBankFile) to get the argument data

Examples

```
## Not run:
up=UniProt.ws()
data=readDBXML(DBXMLFilePath)
relations=getUPKBDBRelations(up,data,c("P02747","P05164"),c("DB00001","DB00006"))

## End(Not run)
```

getUPKBInfo — *Get Proteins from UniProtKB*

Description

Get Proteins from UniProtKB

Usage

```
getUPKBInfo(up, proteins, col)
```

Arguments

up	The UniProt.ws Object
proteins	The list of UniProtKB Proteins ID to be retrieved
col	The list of attributes associated to the UniProtKB Entries to be retrieved

Value

a dataframe containing the protein entries with the selected attributes

Note

Should be preceded by UniProt.ws() to get the UniProt.ws Object To get the list of possible columns, you can call columns(UniProt.ws())

Examples

```
## Not run:
up <- UniProt.ws()
getUPKBInfo(up,c("Q6ZS62","P14384"),c("PROTEIN-NAMES","GO"))

## End(Not run)
```

getUPKBInteractions — *Get the interactions of given proteins from UniProt*

Description

Get the interactions of given proteins from UniProt

Usage

```
getUPKBInteractions(up, proteins)
```

Arguments

up	The UniProt.ws Object
proteins	The list of proteins of which the interactions should be retrieved

Value

A dataframe containing the interactions between the given proteins

Note

Should be preceded by UniProt.ws() to get the UniProt.ws Object

Examples

```
## Not run:
up=UniProt.ws()
interactions=getUPKBInteractions(up,c("P02747","P07204","P00734"))

## End(Not run)
```

---

getUPKBtoDB   *Get UniProt Proteins to DrugBank Drugs relations from UniProt*

Description

Get UniProt Proteins to DrugBank Drugs relations from UniProt

Usage

```
getUPKBtoDB(up, proteinList, drugList)
```

Arguments

`up`	The UniProt.ws Object
`proteinList`	The list of UniProt Ids of the proteins
`drugList`	The ID of the DrugBank drug entry starting with "DB". This argument can be either a string (one drug) or a list of strings (multiple drugs).

Value

A dataframe containing the connections between UniProt proteins and DrugBank drugs retrieved from UniProt

Note

Should be preceded by UniProt.ws() to get the UniProt.ws Object

Examples

```
## Not run:
up=UniProt.ws()
getUPKBtoDB(up,c("P02747","P00734","P07204"),c("DB00001","DB00002"))

## End(Not run)
```

loadDBXML — *Load DrugBank XML file*

Description

Load DrugBank XML file

Usage

```
loadDBXML(file)
```

Arguments

`file`	The path to the DrugBank XML file. This can be downloaded from the DrugBank official Website (drugbank.ca). An account with an institutional e-mail is required.

Value

A dataframe containing the parsed information from DrugBank. This can be used to extract any additional information on the DrugBank entries

Note

This function should be called before using any function to query the DrugBank database. Since the parsing of DrugBank takes time, this function should only be called once.

multipath — *Generate Multipath Graph from General Data*

Description

Generate Multipath Graph from General Data

Usage

```
multipath(
  name = "Multipath",
  up = NA,
  proteinList = NA,
  data = NA,
  drugList = NA
)
```

Arguments

- `name` The name of the graph to be generated
- `up` The Uniprot.ws() object
- `proteinList` The list of proteins of which the interactions should be retrieved
- `data` The dataframe containing the parsed information of DrugBank. This argument can be obtained using the function loadDBXML(DrugBankFile)
- `drugList` The list of DrugBank Ids of the drugs. This argument can be either a string (one drug) or a list of strings (multiple drugs)

Value

A mully graph with the added data

`pathway2Mully` *Build a mully graph from a given pathway*

Description

Build a mully graph from a given pathway

Usage

```
pathway2Mully(biopax, pathwayID)
```

Arguments

- `biopax` The BioPaX object containing the parsed data from an OWL file. This can be obtained using readBiopax(filepath)
- `pathwayID` The ID of the pathway in the biopax object

Value

A mully graph built from the given pathway

Note

This should be preceded by readBiopax(filepath) to obtain the biopax object

Examples

```
## Not run:
biopax=readBiopax(pi3k.owl)
pi3kmully=pathway2mully(biopax,"pathway1")

## End(Not run)
```

pathwayView *Create an empty view*

Description

Create an empty view

Usage

```
pathwayView(g, name)
```

Arguments

g	The input graph
name	The name of the view

Value

An empty view

Examples

```
## Not run:
view=pathwayView(mully("myMully",T),"View1")

## End(Not run)
```

print.pathwayView *Print function*

Description

Print function

Usage

```
## S3 method for class 'pathwayView'
print(x, ...)
```

Arguments

x	The input View to be printed
...	any other parameteres passed to `print`

`undo` — *Undo a modification step in a view*

Description

Undo a modification step in a view

Usage

```
undo(v, stps = 1)
```

Arguments

`v`	The input view
`stps`	The number of steps to undo. This number referes to the number of unique steps' IDs to be removed, i.e. entries of steps in the view with similar stepID count as 1

Value

The view with the undone modifications

`wntpathway` — *Demo function for Wnt Pathway Views*

Description

Demo function for Wnt Pathway Views

Usage

```
wntpathway(file)
```

Arguments

`file`	The link to the Wnt Pathway biopax file

Index

addDBLayer, 2
addStep, 3
addUPKBLayer, 4

downloadPathway, 5

getAllUPKB, 5
getDBCarriers, 6
getDBDrug, 6
getDBDrugInteractions, 7
getDBEnzymes, 8
getDBTargets, 8
getDBtoUPKB, 9
getDBTransporters, 9
getExternalIDs, 10
getPathwayID, 10
getUPKBDBRelations, 11
getUPKBInfo, 12
getUPKBInteractions, 12
getUPKBtoDB, 13

loadDBXML, 14

multipath, 14

pathway2Mully, 15
pathwayView, 16
print.pathwayView, 16

undo, 17

wntpathway, 17
